# The Small Bowel's Big Secret: A Case of Hidden Adenocarcinoma Unmasked by Capsule Endoscopy

**DOI:** 10.7759/cureus.103411

**Published:** 2026-02-11

**Authors:** Shagufta K Kiran, Rafique Hussain, Ali Raza, Hira Gul, Pardeep Maheshwari

**Affiliations:** 1 Internal Medicine, University Hospital Limerick, Limerick, IRL; 2 Pulmonology, University Hospital Limerick, Limerick, IRL; 3 Respiratory Medicine, University Hospital Limerick, Limerick, IRL; 4 Gastroenterology and Hepatology, University Hospital Limerick, Limerick, IRL

**Keywords:** chronic abdominal pain, iron deficiency anaemia, resource limited setting, small bowel adenocarcinoma, small bowel capsule endoscopy

## Abstract

Small bowel adenocarcinoma (SBA) is an uncommon gastrointestinal malignancy that frequently presents with vague or nonspecific symptoms, leading to delayed diagnosis and poor outcomes. Because the small bowel is largely inaccessible to standard endoscopy, many patients endure prolonged abdominal pain, iron deficiency anemia, and weight loss before the underlying pathology is identified. Capsule endoscopy can reveal lesions that are often missed on upper or lower endoscopy and cross-sectional imaging, but its use remains limited in many centers due to issues of availability, cost, or delayed clinical consideration.

We report the case of a 79-year-old man with a history of ischemic heart disease and paroxysmal atrial fibrillation who presented with one year of post-prandial abdominal pain, vomiting, and significant weight loss. Initial investigations, including gastroscopy, colonoscopy, and computed tomography of the abdomen and pelvis, were unrevealing. Capsule endoscopy subsequently identified a small bowel mass, and surgical resection confirmed moderately to poorly differentiated adenocarcinoma with negative margins and no nodal involvement. Following surgery, the patient recovered well and remains symptom-free on follow-up imaging.

This case highlights the diagnostic challenge of SBA and reinforces the importance of considering capsule endoscopy early in patients with persistent, unexplained gastrointestinal symptoms, especially in elderly individuals with iron deficiency anemia. It also underscores the need for wider access to small bowel imaging in resource-limited settings and calls for further research into optimal post-resection surveillance strategies for small bowel malignancies.

## Introduction

Small bowel adenocarcinoma (SBA) is a rare malignancy, representing only 1-3% of all gastrointestinal cancers [1,2]. Diagnosis is often delayed due to its vague presentation, typically intermittent abdominal pain, anemia, or weight loss, and the limited reach of standard endoscopy [3]. Consequently, patients may experience prolonged morbidity before diagnosis, with a significant proportion presenting at advanced stages. Studies have shown that 21-27% of patients have regional lymph node involvement and 32-37% present with distant metastases at the time of diagnosis [4].

These diagnostic challenges are exacerbated in settings where capsule endoscopy, an invaluable tool for evaluating the mid/small bowel, is unavailable, unaffordable, or underutilized. Early consideration of capsule endoscopy in cases of unexplained gastrointestinal bleeding or chronic abdominal pain may improve early detection and survival outcomes [5,6].

## Case presentation

A 79-year-old man with ischemic heart disease (status post percutaneous coronary intervention) and paroxysmal atrial fibrillation on a non-vitamin K antagonist oral anticoagulant presented with a year-long history of post-prandial abdominal pain, occasional vomiting, and significant weight loss.

Laboratory findings revealed iron deficiency anemia with normal rest of the baseline investigations (Table 1).

**Table 1:** Relevant lab investigations of the patient.

Test	Result	Reference Range	Interpretation
Hemoglobin	9.1 g/dL	13.5-16.5 g/dL	Low
Transferrin Saturation	5%	15-45%	Low
Alanine Amino Transferase	9 U/L	10-39 U/L	Normal
Gamma Glutamyl Transferase	14 U/L	10-71 U/L	Normal
Alkaline Phosphatase	117 U/L	40-129 U/L	Normal
Amylase	33 U/L	28-100 U/L	Normal

**Figure 1:**
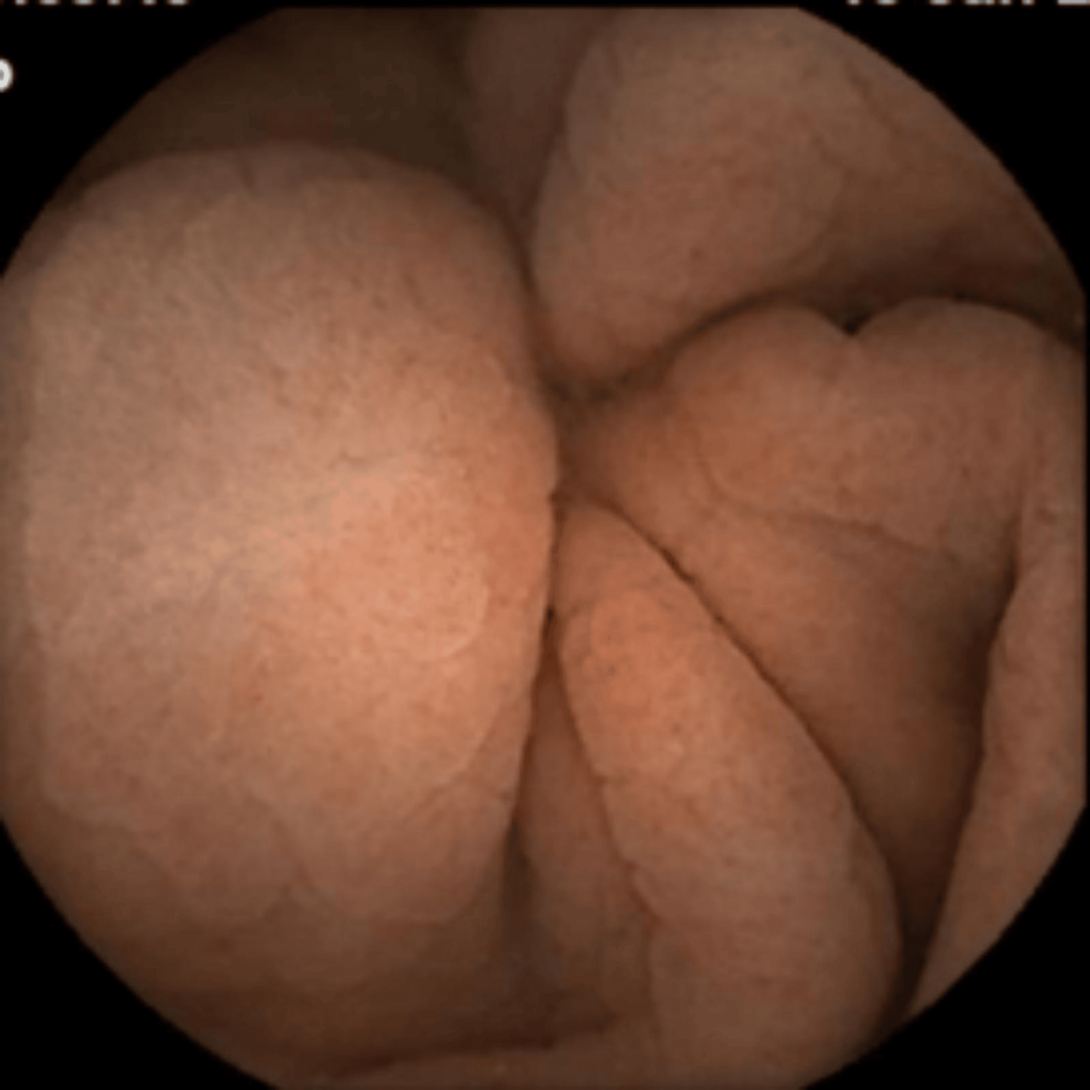
Capsule endoscopy image showing normal small bowel mucosa.

**Figure 2:**
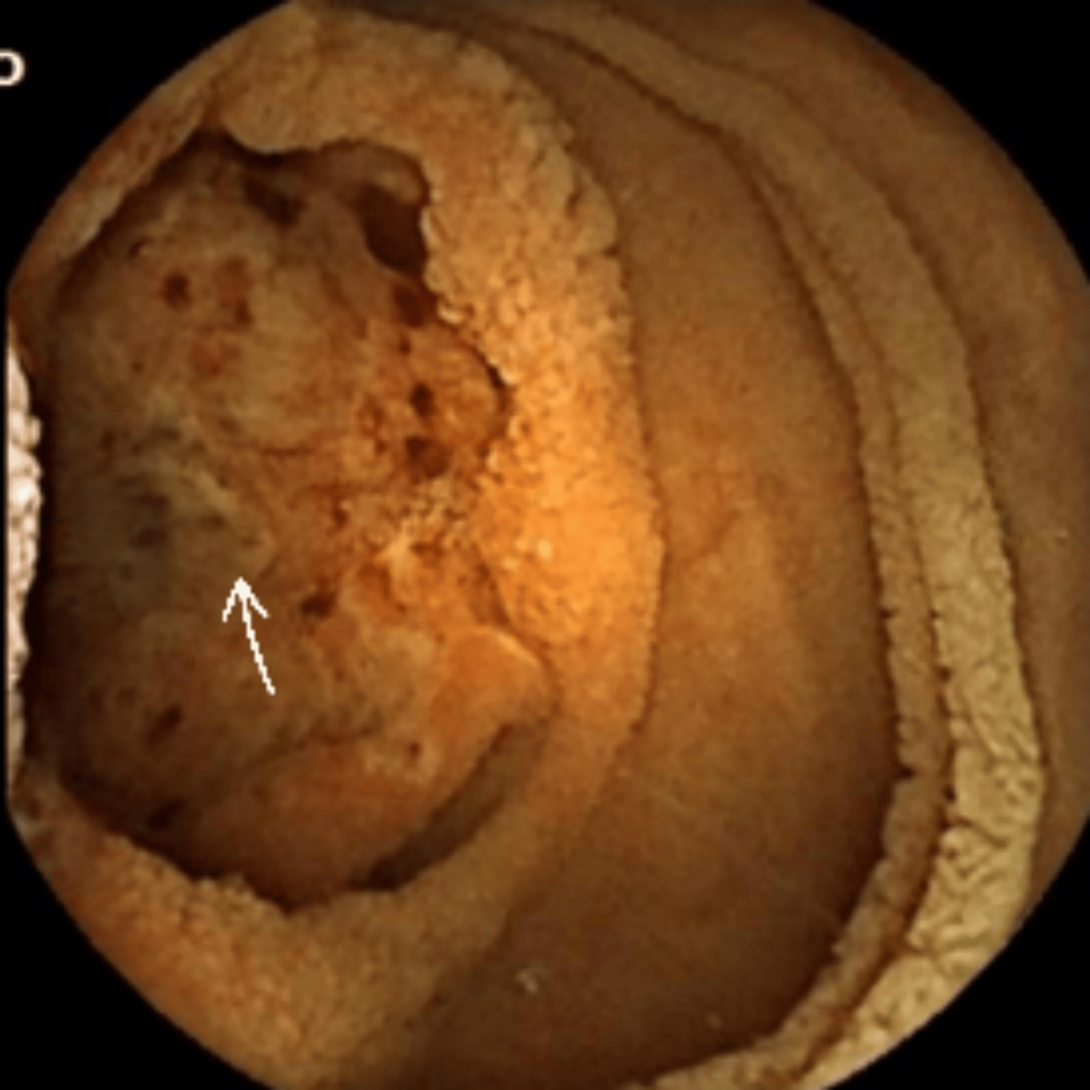
Capsule endoscopy image showing a mass in bowel mucosa with ulceration as denoted by the arrow.

An upper gastrointestinal endoscopy demonstrated mild Esophagitis, while a colonoscopy showed benign colonic polyps and a lipoma. A contrast-enhanced CT scan of the abdomen and pelvis revealed no evidence of malignancy. Prior investigations for bacterial overgrowth and celiac disease were negative. Despite proton pump inhibitors and antispasmodic therapy, his symptoms persisted. Capsule endoscopy revealed a 3 cm small bowel mass, prompting urgent surgical referral (Figures 1-2).

**Figure 3:**
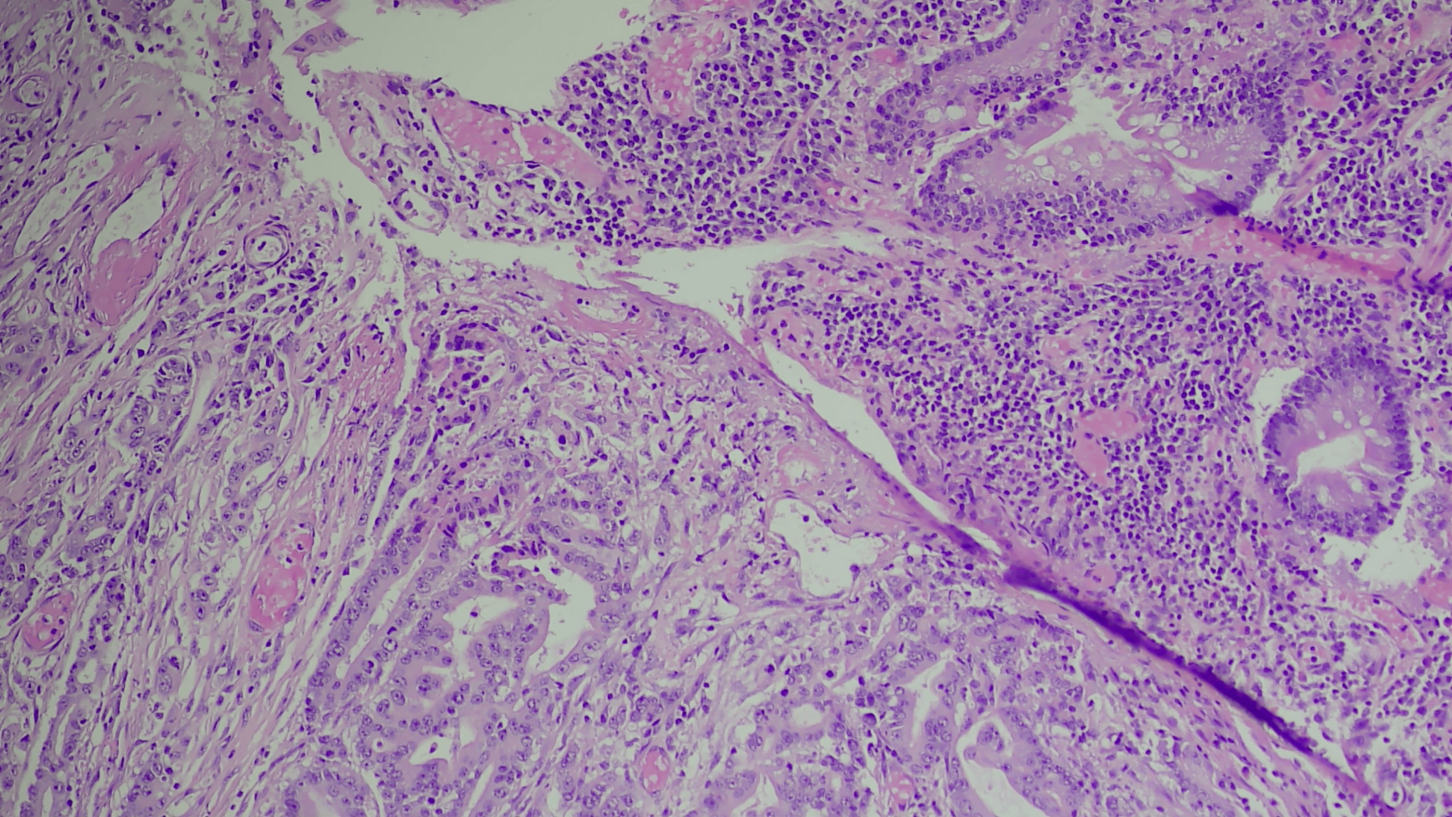
(H&E ×10) Histological examination of the mass showing irregular infiltration of glands/tubules invading through the bowel wall. In the upper right portion of the image, the cells that form more uniform and organized structures, such as the clear glandular lumen, represent normal or non-malignant tissue. These cells have more consistent nuclei and a more regular arrangement compared to the malignant cells. The clusters of cells forming irregular, crowded glands in the lower-left and central parts of the image are consistent with cancer cells, where cells are arranged in a disorderly fashion, the nuclei within these cells are varied in size and shape (pleomorphism), and often appear darker than the surrounding cells (hyperchromatism), which is a result of increased DNA content.

**Figure 4:**
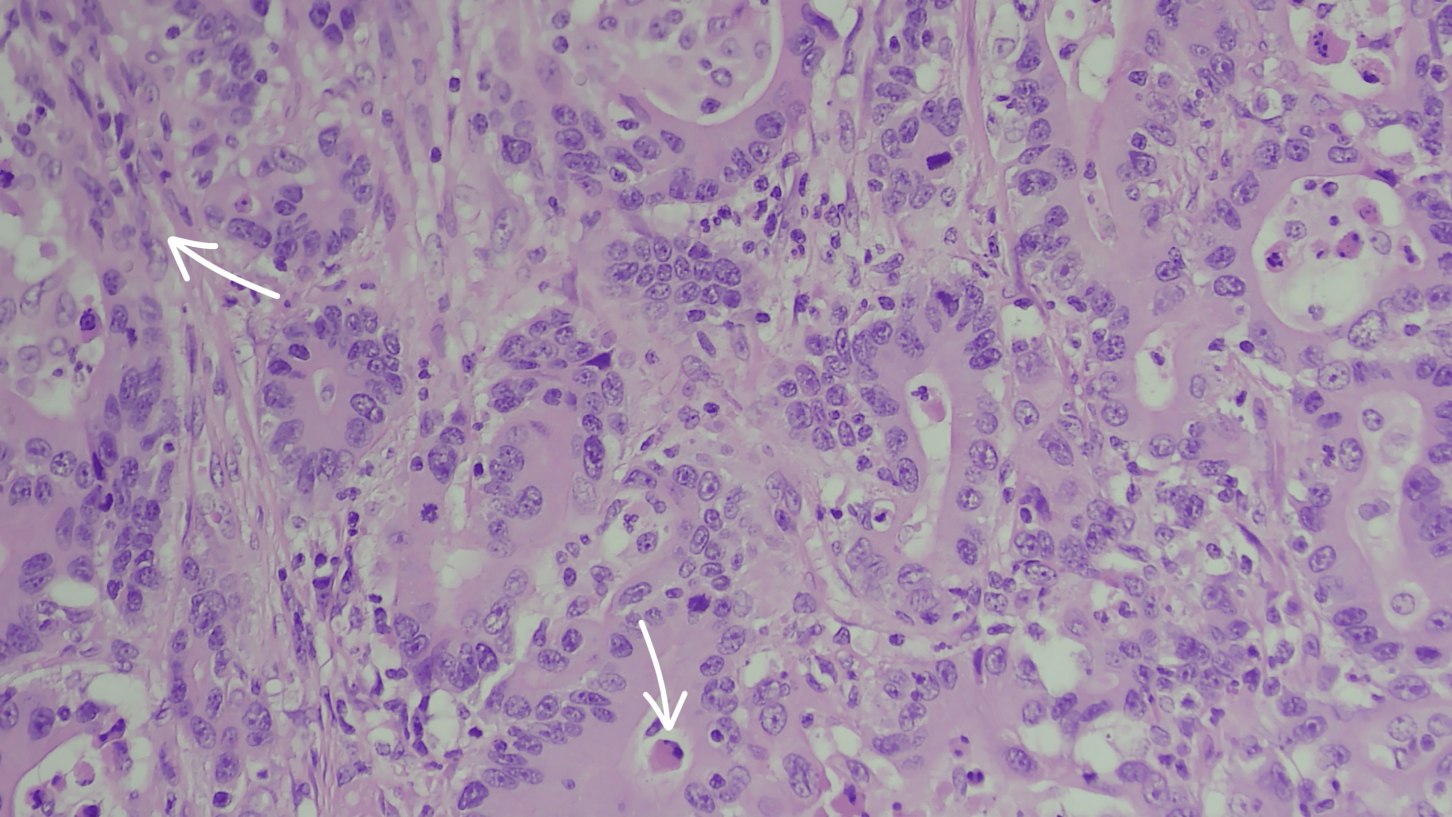
(H&E ×400) This magnified Image from histological examination of the mass shows presence of disorganized structures, irregular placement of nuclei (loss of polarity), cells are not forming the uniform, well-ordered glandular structures typical of healthy tissue as shown by the arrow in top left, presence of signet ring cells as denoted by the central bottom arrow, and the pinkish, fibrous connective tissue surrounding the abnormal glands is known as desmoplasia, a common stromal response to invasive cancer.

Before the elective surgery could be performed, clinical deterioration due to intestinal obstruction necessitated emergency resection. Histopathology confirmed a moderately to poorly differentiated adenocarcinoma of the small bowel, measuring 3 cm, with negative resection margins, no lymphovascular invasion, and 24 lymph nodes negative for metastasis (Figures 3-4).

**Figure 5:**
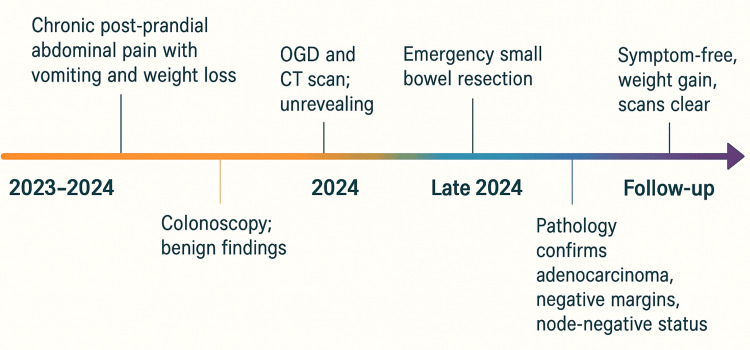
Patient's progress.

Given the absence of high-risk pathological features, surveillance was recommended. Follow-up at multidisciplinary discussion (MDM) confirmed concordant radiological findings and no recurrence. At his latest follow-up, the patient remains symptom-free, has regained weight, and demonstrates no evidence of disease on imaging (Figure 5).

## Discussion

SBA is rare and often diagnosed late due to nonspecific symptoms and limited accessibility of the small bowel to conventional endoscopy [1,2,6]. Iron deficiency anemia and vague abdominal pain may persist despite normal upper and lower endoscopies, reflecting the low sensitivity of standard CT, which detects only about half of small bowel tumors (≈55.8%) with a sensitivity of around 40% for subtle intraluminal lesions [7]. Capsule endoscopy markedly improves diagnostic yield, identifying 79-83% of small bowel tumors, while CT enterography detects 93-94% of tumors when performed with dedicated protocols, demonstrating higher sensitivity for structural lesions but continued value of endoscopy for mucosal evaluation [7,8].

Despite its utility, capsule endoscopy remains underutilized in many settings, contributing to delayed diagnosis and poorer outcomes [9,10]. Prognosis is strongly stage-dependent, with five-year survival exceeding 80% in localized disease compared to ~30-35% overall [2,11]. The absence of standardized post-resection surveillance protocols highlights ongoing uncertainty in long-term management [12].

What this case adds

This case illustrates how SBA can remain undetected despite normal endoscopy and CT imaging and emphasizes the pivotal role of capsule endoscopy in reducing diagnostic delay and facilitating potentially curative intervention.

Patient perspective

The patient reports significant improvement in overall health and quality of life, weight gain, and complete resolution of symptoms. He expressed gratitude for the persistence shown in pursuing a definitive diagnosis after multiple inconclusive investigations.

## Conclusions

Early use of capsule endoscopy is crucial for patients with persistent, unexplained gastrointestinal symptoms, particularly iron deficiency anemia, as it can significantly reduce diagnostic delays and improve outcomes. Expanding access to capsule endoscopy, including in resource-limited settings, remains a key priority.

There is also an unmet need for research to establish standardized post-resection surveillance, as current guidelines are limited. Timely diagnosis, appropriate surgical intervention, and structured follow-up remain essential for optimizing prognosis in SBA.
